# The Zinc Concentration in the Diet and the Length of the Feeding Period Affect the Methylation Status of the *ZIP4* Zinc Transporter Gene in Piglets

**DOI:** 10.1371/journal.pone.0143098

**Published:** 2015-11-23

**Authors:** Diana Karweina, Susanne Kreuzer-Redmer, Uwe Müller, Tobias Franken, Robert Pieper, Udo Baron, Sven Olek, Jürgen Zentek, Gudrun A. Brockmann

**Affiliations:** 1 Breeding Biology and Molecular Genetics, Albrecht Daniel Thaer-Institute of Agri- and Horticulture, Faculty of Life Sciences, Humboldt-Universität zu Berlin, Berlin, Germany; 2 Institute of Animal Nutrition, Department of Veterinary Medicine, Freie Universität Berlin, Berlin, Germany; 3 Epiontis GmbH, Berlin, Germany; University of Navarra, SPAIN

## Abstract

High doses of zinc oxide are commonly used in weaned pig diets to improve performance and health. Recent reports show that this may also lead to an imbalanced zinc homeostasis in the animal. For a better understanding of the regulatory mechanisms of different zinc intakes, we performed a feeding experiment to assess potential epigenetic regulation of the *ZIP4* gene expression via DNA methylation in the small intestine of piglets. Fifty-four piglets were fed diets with 57 (LZn), 164 (NZn) or 2,425 (HZn) mg Zn/kg feed for one or four weeks. The *ZIP4* expression data provided significant evidence for counter-regulation of zinc absorption with higher dietary zinc concentrations. The CpG +735 in the second exon had a 56% higher methylation in the HZn group compared to the others after one week of feeding (8.0·10^-4^ < *p* < 0.035); the methylation of this CpG was strongly negatively associated with the expression of the long *ZIP4* transcripts (*p* < 0.007). In the LZn and NZn diets, the expression of the long *ZIP4* transcripts were lower after four *vs*. one week of feeding (2.9·10^-4^ < *p* < 0.017). The strongest switch leading to high DNA methylation in nearly all analysed regions was dependent on feeding duration or age in all diet groups (3.7·10^-10^ < *p* < 0.099). The data suggest that DNA methylation serves as a fine-tuning mechanism of *ZIP4* gene regulation to maintain zinc homeostasis. Methylation of the *ZIP4* gene may play a minor role in the response to very high dietary zinc concentration, but may affect binding of alternate zinc-responsive transcription factors.

## Introduction

Weaning of piglets is a stressful phase that is often accompanied by diarrhoea and reduced growth performance [1–5]. Since the ban of antibiotic feed additives in the European Union [6] diverse other feed additives have been proposed to improve gut health in pigs [7] including trace elements such as copper and zinc [8].

Zinc is an essential trace element for all species. It is a structural component and acts as a catalytic cofactor of about 3,000 proteins [9]. Moreover, zinc and zinc-dependent proteins are essential modulators for the epigenome since they are involved in several pathways relevant for epigenetic regulation and modifications [10,11]. Zinc has a specific role for immunity, deficiencies are associated with growth retardation, immune dysfunction, and infertility [12,13]. Although the recommendations for dietary zinc levels vary from 50 to 100 mg zinc/kg feed [14,15], several studies in pigs demonstrated an improvement of growth performance and a reduction of post weaning diarrhoea at concentrations from 1,000 to 3,000 mg zinc oxide per kg feed [16]. However, such high zinc supplementation is accompanied with increased organ accumulation [17–19] and may induce detrimental effects in organs such as liver [20] and pancreas [21] indicating an outbalanced zinc homeostasis.

The zinc transporter 4 (ZIP4; alias solute carrier family member A4, SLC39A4) is the most important transporter for the uptake of zinc from the lumen of the small intestine [22]. The active transporter is located at the apical surface of the enterocytes in the intestinal epithelium [22,23]. *ZIP4* is regulated by zinc intake and mainly promotes the influx of zinc into the cells [23]. The expression of *ZIP4* as well as the translocation of the protein to the cell membrane seems to be regulated directly by the local zinc concentration in the gut [22,23]. Previous experiments with piglets fed with high zinc amounts of 2,500 mg zinc per kg feed have shown that the expression of *ZIP4* was down-regulated, likely to protect the cells from toxic effects [18]. It is still not clear by which molecular mechanisms zinc contributes to such transcriptional down-regulation of the *ZIP4* gene.

Since the DNA sequence of the *ZIP4* gene features a high amount of so called CpG sites we expected a regulative role of DNA methylation in the transcriptional activity of the *ZIP4* gene. CpGs are methylation sensitive cytosines in the context of CpG dinucleotides within one gDNA strand (CpG, CpG site) at which DNA methylation occurs. Such CpG sites can cumulate in regions called CpG islands with more than 200 bases and a G+C content of at least 50% [24], whereas CpG island shores (CpG shores) are regions of lower CpG density that lie in close proximity (~2 kb) of CpG islands [25].

In the present study, we analysed the DNA methylation pattern of the *ZIP4* gene and its transcriptional activity in jejunal epithelial cells from the small intestine of piglets fed with three different zinc diets for one or four weeks. Our aim was to test if epigenetic modifications in putative regulatory regions of the *ZIP4* gene contribute to the transcriptional regulation of this gene in addition to direct effects of the dietary zinc concentration.

## Materials and Methods

### Animals and housing

All procedures involving piglet handling and treatments were approved by the local state office of occupational health and technical safety ‘Landesamt für Gesundheit und Soziales Berlin’ (LaGeSo Reg. Nr. 0347/09).

A total of 54 purebred German Landrace piglets (born and raised at the Institute of Animal Nutrition, Freie Universität Berlin, Germany) were used in the study. They were housed in commercial flat deck pens (n = 2/pen) in stainless steel framings. After weaning at the age of 26 ± 1 days, the piglets, which are from the identical set of animals previously described [18], were randomly distributed into three feeding groups with eighteen piglets each balanced for litter, gender and body weight (BW) (mean-BW: 7.7 ± 1.1 kg). Piglets in each group received one of three experimental diets with zinc levels of 57 (low dietary zinc, LZn), 164 (normal dietary zinc, NZn), and 2,425 (high dietary zinc, HZn) mg Zn/kg feed. All pigs had *ad libitum* access to water and feed. Room temperature was kept at 26°C with stabling, and was incrementally decreased to 22°C within the first week after weaning with constant humidity. The light program warranted a 16 hours light and an 8 hours dark phase. BW and feed intake were recorded for every week of experiments and were described and reported previously [18]. No clinical signs of disease or impaired health were determined. No antibiotics or other medications were administered before or during the experiments. After zinc feeding periods of one or four weeks (32 ± 2 and 53 ± 2 days of piglet age, respectively), eight to ten pigs per group were randomly selected and killed for tissue sampling.

### Diets

From 12 days of age, piglets had access to a non-medicated prestarter diet which was formulated to meet the requirements of piglets with an average BW of 5–10 kg [14]. After weaning, piglets received a mash starter diet based on wheat, barley, and soybean meal with different zinc concentrations (Table 1). Zinc concentration of starter diet was adjusted to 57, 164 or 2,425 mg Zn/kg feed by supplementation of corn starch with analytical grade zinc oxide (Sigma Aldrich, 204951). The main methyl donors of the diet were Methionine, Riboflavin, Vitamin B6, Vitamin B12, Folic acid, and Choline chloride (Table 1).

**Table 1:** Ingredients and calculated chemical composition of the diets.

Ingredients	*g/kg feed*	Chemical composition	*g/kg feed*
Wheat	380.0	Starch	375.0
Barley	300.0	Crude protein	194.0
Soybean meal	232.0	Moisture	120.0
Monocalcium phosphate	20.0	Crude ash	81.0
Limestone	20.0	Crude fibre	36.0
Soy oil	17.5	Ether extract	34.0
Mineral & Vitamin Premix^1^	15.0	Metabolisable Energy (MJ/kg)	13.0
Corn starch/zinc oxide^2^	10.0	Lysine	11.7
Lysine HCL	2.5	Calcium	11.1
Salt	2.0	Phosphorus	8.4
D/L-Methionine	1.0	Threonine	7.2
		Methionine	4.0
		Sodium	3.0
		Tryptophan	2.4
		Magnesium	2.1
		Iron	0.309
		Manganese	0.041
		Zinc^3^	0.035
		Copper	0.008

^1^ Mineral and Vitamin Premix (Spezialfutter Neuruppin Ltd., Neuruppin, Germany), containing per kg dry matter: 130 g Sodium (as sodium chloride), 55 g Magnesium (as magnesium oxide), 210 mg Retinol, 3 mg Vitamin D3, 8 g DL-a-Tocopherol, 300 mg Menadione, 250 mg Thiamine, 250 mg Riboflavine, 400 mg Vitamin B6, 2 mg Vitamin B12, 2,5 g Nicotinic acid, 100 mg Folic acid, 25 mg Biotin, 1 g Pantothenate, 80 g Choline chloride, 5 g Iron (as Iron-(II)-carbonate), 1 g Copper (as Copper-(II)-sulfate), 6 g Manganese (as Manganese-(II)-oxide), 45 mg Iodine (as Calcium-iodate), 35 mg Selenium (as Sodium-selenite).

^2^ Corn starch in the basal diet was partially replaced in the three diets containing 50, 150, and 2500 mg/kg zinc with analytical grade zinc oxide (Sigma Aldrich, Deisenhofen, Germany) to adjust for the zinc level.

^3^ Analyzed concentration of zinc in the basal diet without ZnO supplementation. The experimental diets contained zinc at 57, 164, and 2,425 mg/kg, respectively.

### Euthanasia and sampling

Piglets were sedated with 20 mg/kg BW of ketamine hydrochloride (Ursotamin®, Serumwerk Bernburg AG, PZN 07005294) and 2 mg/kg BW of azaperone (Stresnil®, Jansen-Cilag, PZN 02002032) prior to euthanasia with intracardial injection of 10 mg/kg BW of tetracaine hydrochloride, mebezonium iodide and embutramide (T61®, Intervet, PZN 03814039). Following euthanasia and a midline abdominal incision, the pyloric valve and ileo-cecal junction were clamped and the entire intestinal tract was removed from the peritoneum. Small intestine was dissected from the large intestine at the ileo-cecal junction and both segments were dissected from the mesentery. Digesta, collected from the distal jejunum for zinc concentration measurements and a tissue section (10 cm) for transcription and methylation analysis, obtained from the mid jejunum, were taken, immediately snap-frozen in liquid nitrogen, and stored at -80°C until further analyses.

### Determination of zinc concentration

For the determination of the zinc concentration in the feed and the digesta, samples were hydrolysed in concentrated hydrochloric acid after freeze-drying and incinerated at 600°C for 8 h. Afterwards, zinc concentration was determined via atomic absorption spectrometry in an AAS vario 6 spectrometer (Analytik Jena, Jena, Germany) as described in detail [21].

### RNA extraction and determination of gene expression

The jejunal tissue samples were homogenized in liquid nitrogen using pestle and mortar. Total RNA was extracted from 30 mg of grounded jejunal tissue using the NucleoSpin® RNAII kit (Marchery-Nagel GmbH Co. KG, 740955). Concentration of the extracts was determined by measuring absorbance at 260 nm (NanoDrop Technologies, Inc., Wilmington, USA). Quality and purity was checked on a BioAnalyzer (Agilent 2100, Agilent, Waldbronn, Germany) as well as per gel electrophoresis. Only RNA samples with a clear and dominant 18S to 28S ribosomal band ratio (conventional agarose gel electrophoresis) and with an absorbance ratio A_260/280_ of ~2.0 (Nanodrop) or a RNA integrity number of at least 7.0 (BioAnalyzer) was considered for further analyses. Afterwards, 1 μg of RNA was used for cDNA synthesis using the two-step qRT-PCR SuperScript® VILO™ cDNA Synthesis Kit (Life Technologies, 11754–050). Each reaction consisted of 2.5 μl SuperScript® VILO™ Master Mix, 1 μg RNA and DEPC-treated water to 15 μl total reaction volume. The reverse transcriptase reaction was performed on a Thermocycler (Biometra, Göttingen, Germany) with the following conditions: First, incubation at 25°C for 10 min, then reverse transcription at 42°C for 60 min, followed by termination of reaction at 85°C for 5 min.

Expression of the zinc transporter *ZIP4* was examined on the transcripts ZIP4-201, ZIP4-001, and ZIP4-004 (S1 Fig). The long transcripts ZIP4-201 and ZIP4-001 contain all 12 exons of the *ZIP4* gene, however ZIP4-001 has a shortened exon 1 with a back shifted transcription start site (TSS). The transcript ZIP4-004 contains the last three exons 10 to 12 with an elongated exon 10 beginning at the end of intron 9. Expression of 60S ribosomal protein L19 (*RPL19*) and TATA box-binding protein (*TBP*) did not vary due to the zinc treatment of the piglets and were used as stable house-keeping genes for normalization of expression data. Primers of the *ZIP4* transcripts were designed exon-boundaries overlapping based on published sequences of the pig (Ensembl, Genome assembly: Sscrofa10.2, [GCA_000003025.4]) using the online primer design tool Primer3Plus (http://www.bioinformatics.nl/cgi-bin/primer3plus/primer3plus.cgi) [26,27] whereas primers of house-keeping genes had already been published (Table 2). Both, the long transcripts ZIP4-201 and ZIP4-001 were undistinguishable during transcript expression analysis and were measured together with the same primer pair.

**Table 2:** List of RT-qPCR primers.

Target	Ensembl-ID^2^	Primer sequences (5´ to 3´)	Reference
ZIP4-201^1^	ENSSSCT00000006485^3^	ACGTCATCCAGACCTTCCTG	This study
		TTTGGGTCCTCTGGGTCTG	
ZIP4-001^1^	ENSSSCT00000035628	ACGTCATCCAGACCTTCCTG	This study
		TTTGGGTCCTCTGGGTCTG	
ZIP4-004	ENSSSCT00000032816	AGGGCCAGGCGGATG	This study
		GCGAAGTCCCCCAGCTC	
*RPL19*	ENSSSCT00000019062	AACTCCCGTCAGCAGATCC	[28]
		AGTACCCTTCCGCTTACCG	
*TBP*	ENSSSCT00000029370	AACAGTTCAGTAGTTATGAGCCAGA	[28,29]
		AGATGTTCTCAAACGCTTCG	

^1^ Both transcripts ZIP4-201 and Zip4-001 include all 12 exons of the gene, but they differ in their transcription start sites; the given primer pair amplifies both transcripts and does not discriminate them.

^2^ Genome assembly: Sscrofa10.2 (GCA_000003025.4).

^3^ http://www.ensembl.org/Sus_scrofa/Transcript/Summary?db=core;g=ENSSSCG00000005905;r=4:464087-469224;t=ENSSSCT00000006485

Quantitative real-time PCR (RT-qPCR) was performed using the SYBR® Select Master Mix (Life Technologies, 4472920). Each RT-qPCR reaction consisted of 2 μl diluted cDNA (5 ng/μl), 5 μl SYBR® Select Master Mix, 0.3 μl each of 10 μM gene- or transcript-specific forward and reverse primers and 2.4 μl RNase-free water to a total reaction volume of 10 μl. The RT-qPCR was performed on a ViiA™ 7 qPCR-System (Life Technologies, Carlsbad, USA) with cycling conditions as follows: One cycle for enzyme mixture activation at 50°C for 2 min, one cycle of initial denaturation at 95°C for 2 min, followed by 40 cycles of a denaturation step at 95°C for 15 s and an annealing and extension step at 60°C for 1 min. Afterwards, a melting curve analysis was performed in every reaction well to check for specificity of primers. All primer sets were initially validated for single amplicon generation and standard curves were tested prior to analysis. Only primer pairs with efficiency rates between 90% and 110% were considered for gene expression analyses. Melting curves and PCR efficiency were used as standard quality criteria for each RT-qPCR run.

Target transcripts were measured in triplicates and house-keeping genes in duplicates on 384 well plates. To allow comparison of PCR reactions performed on different plates, mean values of triplicate and duplicate Ct values of every gene or transcript for every sample were first corrected. In doing so, the mean value of all Ct values of a standard sample was subtracted from the mean Ct values of every gene or transcript for every sample. The standard sample consisted of pooled cDNA from six animals, measured for every gene or transcript on every well plate. For every target transcript in every sample, the difference between the corrected mean Ct value of the target transcript and the corrected mean Ct value of the two housekeeping genes was calculated to get a normalized arbitrary ΔCt value. To get a ΔΔCt value, the calculated ΔCt value was then calibrated externally with the arithmetic mean of ΔCt values of transcripts ZIP4-201 and ZIP4-001 of all piglets fed NZn diet for one week. Afterwards 2^-ΔΔCt^-value was calculated for every ΔΔCt value and used for statistical comparison [30].

### DNA extraction and bisulfite sequencing

Genomic DNA (gDNA) was extracted from 25 mg of the same grounded jejunal tissue that was used for RNA isolation using the NucleoSpin® Tissue kit (Marchery-Nagel GmbH Co. KG, 740952). Concentration, quality and purity were determined by measuring absorbance at 260 and 280 nm (NanoDrop Technologies, Inc., Wilmington, USA) and per gel electrophoresis.

For sodium bisulfite conversion 1 μg of gDNA was utilized using the EZ DNA Methylation-Gold™ kit (Zymo Research Europe, D5005). During bisulfite treatment, all unmethylated cytosines of the gDNA were converted to thymines whereas methylated cytosines were not changed. The conversion reaction was performed on a Thermocycler (Biometra, Göttingen, Germany) with cycling conditions as follows: First denaturation at 98°C for 10 min, followed by incubation at 64°C for 2.5 hours. After kit specific column-based purification, bisulfite-modified gDNA (biDNA) was amplified using the Zymo Taq™ DNA Polymerase system (Zymo Research Europe, E2001). Each reaction consisted of 50–100 ng biDNA, 15 μl 2x reaction buffer, 3 μl 10 mM dNTPs, 1.5 μl each of 10 μM *ZIP4* gene region specific forward and reverse primers (Table 3), 0.24 μl 5 U/μl Zymo Taq™ DNA Polymerase and RNase-free water to a total reaction volume of 30 μl. The amplification reaction was performed on a Thermocycler (Biometra, Göttingen, Germany) with following cycling conditions: First one cycle of initial denaturation at 95°C for 10 min, followed by 40 cycles of a denaturation step at 95°C for 30 sec, an annealing step at 55°C for 40 sec and an extension step at 72°C for 1 min, ending with one cycle of final extension at 72°C for 7 min.

**Table 3:** Amplicons, assays, and primers used for methylation status analysis at the *ZIP4* gene locus.

CpG Region	*ZIP4* gene region	*#* CpGs	Location from TSS [Fragment length] *(bp)*	Primer sequences (5´ to 3´)	Sequencing method
Amplicon 1	5´-Region	10	-2236 to -1758 [478]	GATTTTAGTGGAGAGGGTTTTT	Sanger
				CCTCCCTTTTCTACATTCCT	
Assay 1	5´-Region	6	-394 to -120 [274]	TTGTTTTTTGGAATGGTTTGTAGTAA	Pyro
				TACCCrCrAAACCCTATAATTAAC(Bio)	
				Seq 1: GAGTGGGGTAGGGGG	
				Seq 2: TTTAGGAGGGGTTTAGTTTT	
Assay 2	Part of 5´-Region, Exon 1 and	30	-186 to +206 [392]	GGAGGATTTGTyGTTGGAATGGTTAA	Pyro
	Intron 1			CCCrTCrAATAAAACCTTACCTT(Bio)	
				Seq 3: TTTATGTAGTTTGTAGTTAT	
				Seq 4: GTTGGAATGGTTAATTT	
				Seq 5: GTAGTATGGTTGGTT	
				Seq 6: GTTTTTAGGTTAAGG	
				Seq 7: GTTTGTTAAATATATTGG	
				Seq 8: TTGGTTGTGTTGTTGGTTTT	
				Seq 9: GGGTAAGGTTYGGAG	
Amplicon 2	Intron 1	17	+177 to +545 [368]	TGTGGAAAGGTAAGGTTTTATT	Sanger
				ACCACCATCCTCAAACTCTAT	
Amplicon 3	Part of Exon 2 and Intron 2	18	+674 to +1129 [455]	GGTAGGTTTGAGAAGTTAGGATT	Sanger
				CCTATAACCCCAAAAACTCTCT	

Abbreviations: # CpGs, number of CpGs analysed in this fragment; TSS, transcriptional start site; Bio, biotinylated; Seq, sequencing primer.

For the quantitative analysis of differential methylation at CpG sites three amplicons in putative regulatory regions of the *ZIP4* gene (Table 3) were examined. Design of the three amplicons to analyse differential DNA methylation was subjected to the following criteria: 1. Positioning of amplicons in regulatory gene regions upstream and downstream of the TSS as well as in CpG shores. 2. Consideration of highly evolutionarily conserved non-coding regions among mammals, which are probably associated with regulatory functions. 3. Verification of sequence composition of amplicons for CpG density (10–20 CpGs per 500 bp) and bisulfite conversion-limited modification to avoid sequence break-offs and artefacts. Amplicons were amplified by PCR, isolated on a 2% agarose gel followed by excision of PCR bands from the gel and purified by using the standard protocol of the GeneJET™ Gel Extraction KIT (Life Technologies, K0691). For subsequent Sanger sequencing, PCR BigDye® Terminator v1.1 Cycle Sequencing Kit (Life Technologies, 4337449) was used. Sequencing reactions were loaded on an ABI Sequencer 310 (Life Technologies, Carlsbad, USA) with cycling conditions as follows: 30 cycles of 95°C for 5 sec and 50°C for 4 min. Each sequencing reaction consisted of 2 ng/100 bp PCR-fragment, 1.0 μl BigDye® Terminator v1.1 Ready Reaction Mix, 1.5 μl BigDye® Sequencing Buffer, 1 μl 5 μM amplicon specific forward or reverse primer and RNase-free water to a total reaction volume of 10 μl. Quantification of methylation was calculated on the basis of the sequencing electropherograms by using ESME software [31] (Epiontis GmbH, Berlin, Germany). The algorithm of ESME uses the four-dye trace files of the sequencing reaction of the PCR product obtained with the biDNA and quantifies the methylation for individual cytosine positions after an alignment with the genomic reference sequence, signal normalization and estimation of effectiveness of bisulfite treatment.

The methylation status of CpGs in the critical region at and around the TSS of the *ZIP4* gene was quantified via pyrosequencing (Table 3). Since this region is rich of repetitive elements, designing of an amplicon as described above was impossible. For pyrosequencing, two assay fragments and nine different sequencing primers were designed using the PSQ 96 MA Software 2.1 (Biotage, Uppsala, Sweden). The pyrosequencing reaction of biDNA required in a first step the conversion of the PCR product to a single-stranded DNA template. This was done with a biotinylated primer. Subsequently, the pyrosequencing reaction was performed on a PSQ 96 MA Pyrosequencer (Biotage, Uppsala, Sweden) using Biotage and PyroMark buffer systems (Qiagen, 979006–979009) according to the customers’ protocols with the site-specific sequencing primer (Table 3).

### *In silico* analysis of transcription factor binding sites

For the analysis of transcription factor binding sites that are potentially affected through differentially methylated CpGs in putative regulatory regions of the porcine *ZIP4* gene, we used ConSite (http://consite.genereg.net/cgi-bin/consite) [32]. We used the set of mammalian transcription factors as model since a porcine model does not exist.

### Structure analysis and protein prediction for *ZIP4*

To assess the configuration of the *ZIP4* gene regarding repetitive elements, RepeatMasker was used (http://www.repeatmasker.org/cgi-bin/WEBRepeatMasker) [33]. The structure prediction of the *ZIP4* transcripts ZIP4-201 and ZIP4-004 was done with the free online tools: Phyre2 (http://www.sbg.bio.ic.ac.uk/phyre2/html/page.cgi?id=index) [34], RaptorX (http://raptorx.uchicago.edu/StructurePrediction/predict/) [35], and I-TASSER (http://zhanglab.ccmb.med.umich.edu/I-TASSER/) [36].

### Statistical analysis

Gene expression patterns in the epithelium of the small intestine and zinc concentrations in the digesta were analysed by univariate variance analysis (ANOVA), F-test for equality of variances and bilateral Student’s t-test for differences between feeding groups, respectively. Data were tested for outlier values by Dean-Dixon-test [37] when *p* < 0.05. Data are given as means ± SD unless otherwise stated.

The influence of methylation on the expression of *ZIP4* transcripts was analysed for the feeding period of one and four weeks applying the following model and using the mixed procedure PROC MIXED in SAS (SAS Version 9.4, SAS Institute Inc., Cary, USA):

$${\text{Y}}_{\text{ijkl}}=\;\mu \;+\;{\text{trt}}_{\text{i}}\;+\;{\text{Sex}}_{\text{j}}+\;{\text{MetCpG}}_{\text{k}}+\;{\text{Fam}}_{\text{l}}+\;{\text{e}}_{\text{ijkl}}$$ (1)

where Y_ijkl_ is the level of gene expression of animal_ijkl_, μ is the overall expression mean, trt_i_ is the fixed effect of the zinc treatment group (i = 1-3), Sex_j_ is the fixed effect of sex (k = 1-2), MetCpG_k_ is the methylation level of a selected CpG, Fam_l_ is the random effect of the family (animals sire-dam combination), and e_ijkl_ the random residual error. For the analysis of the treatment effects on the methylation of a CpG, the model was reduced to treatment, sex and family effects, whereas Y_ijkl_ was the methylation level of a selected CpG of animal_ijkl_. For controlling the familywise error rate in pairwise comparisons of group means the Bonferroni adjustment was used. Data are given as least square means (LSMeans) ± SE unless otherwise stated.

Spearman’s correlation coefficients (r) were calculated for the correlation between the individual zinc concentrations in the digesta of the small intestine and the respective relative gene expression of the *ZIP4* transcripts of the jejunal epithelium and between the methylation percentage of a single CpG or groups of CpGs and the expression of the *ZIP4* transcripts ZIP4-201 and ZIP4-001 in the jejunal epithelium.

For all analyses, differences at *p* < 0.01 and *p* < 0.05 were considered as highly significant and significant, respectively. Differences at *p* < 0.1 were considered as varying by trend.

## Results

### High zinc concentrations in the diet led to high zinc concentrations in the digesta

The zinc amount in the digesta of the small intestine was measured in the piglets fed with LZn, NZn, and HZn diets for one and four weeks. Zinc concentrations in the diet were found to be proportional to zinc concentrations in the digesta of the small intestine after one as well as after four weeks of feeding (Table 4). In particular, feeding of the HZn diet for one and four weeks correlated with 9.3 times higher total zinc concentrations in the digesta compared to the NZn feeding group (*p* = 0.001). Four weeks of feeding tended to reduce zinc concentrations in the digesta in the LZn (*p* = 0.08) and NZn (*p* = 0.09) groups compared to one week feeding.

**Table 4:** Zinc concentration in piglet’s jejunal digesta fed different zinc diets for one and four weeks.

	Zinc concentration, *mg/kg dry matter*									
	LZn			NZn			HZn			
Feeding period, *weeks*	*mean*	*SD*	*n*	*mean*	*SD*	*n*	*mean*	*SD*	*n*	ANOVA *p*-value _diet_
1	790.0^*a*^ ^,^ ^*A*^	311.8	5	1048.0^*a*^ ^,^ ^*A*^	186.9	4	9727.9^*b*^	1534.6	4	4.4·10^−8^
4	516.7^*a*^ ^,^ ^*B*^	138.7	6	680.2^*a*^ ^,^ ^*B*^	349.4	6	6339.4^*b*^	3762.9	10	2.4·10^−4^

Abbreviations: LZn, low dietary zinc = 57 mg zinc/kg feed; NZn, normal dietary zinc = 164 mg zinc/kg feed; HZn, high dietary zinc = 2,425 mg zinc/kg feed.

^a,b^Labelled means with different letters in a row are significantly different (*p* < 0.01).

^*A*,*B*^Labelled means with different letters in a column are different by trend (*p* < 0.1).

### Feeding high dietary zinc concentrations reduced the relative expression of all *ZIP4* transcripts

**Fig 1:**
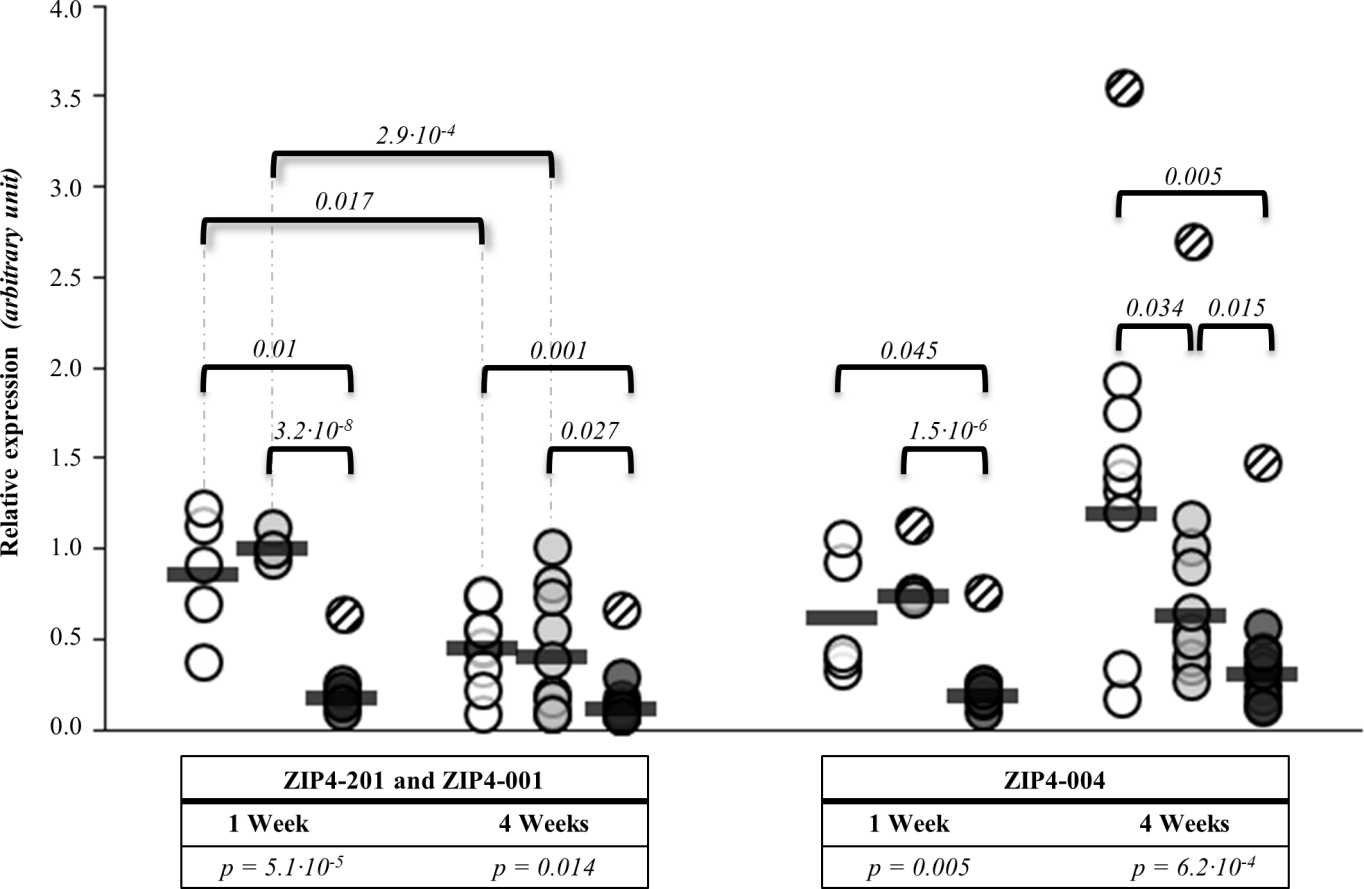
Influence of zinc on *ZIP4* expression in jejunal epithelium after one and four weeks treatment.

One as well as four weeks of HZn feeding was correlated with a lower expression of the transcripts ZIP4-201 and ZIP4-001 (long transcripts, differing in the TSS) and ZIP4-004 (short transcript) by an average of 73% compared to the other feeding groups (*p* < 0.014) (Fig 1, S1 Table). Four weeks of feeding LZn (*p* = 0.017) and NZn (*p* = 2.9·10^-4^) diets led to down-regulation of the expression of ZIP4-201 and ZIP4-001 transcripts compared to one week feeding. We observed that the relationship of decreased mRNA transcript with increased dietary zinc would hold true for the short transcript, ZIP4-004, after four weeks of feeding (*p* = 6.2·10^-4^).

Shown is the relative mRNA expression of the *ZIP4* gene transcripts ZIP4-201, ZIP4-001, and ZIP4-004 in the jejunum of piglets fed three different zinc concentrations (LZn, NZn, and HZn) for one or four weeks. White circle: LZn (low dietary zinc) = 57 mg zinc/kg feed, n _Week1_ = 5, n _Week4_ = 9; grey circle: NZn (normal dietary zinc) = 164 mg zinc/kg feed, n _Week1_ = 4, n _Week4_ = 10; black circle: HZn (high dietary zinc) = 2,425 mg zinc/kg feed, n _Week1_ = 7, n _Week4_ = 10. All expression values of the transcripts are relative to the mean of the ΔCt values of the NZn group after one week NZn diet feeding. Striped data points symbolize outlier values (see Material and Methods part). ANOVA *p*-values below the x-axis present the influence of the zinc feeding group on the expression of transcripts. Pairwise *p*-values above data points present significance of Student’s t-test.

**Fig 2:**
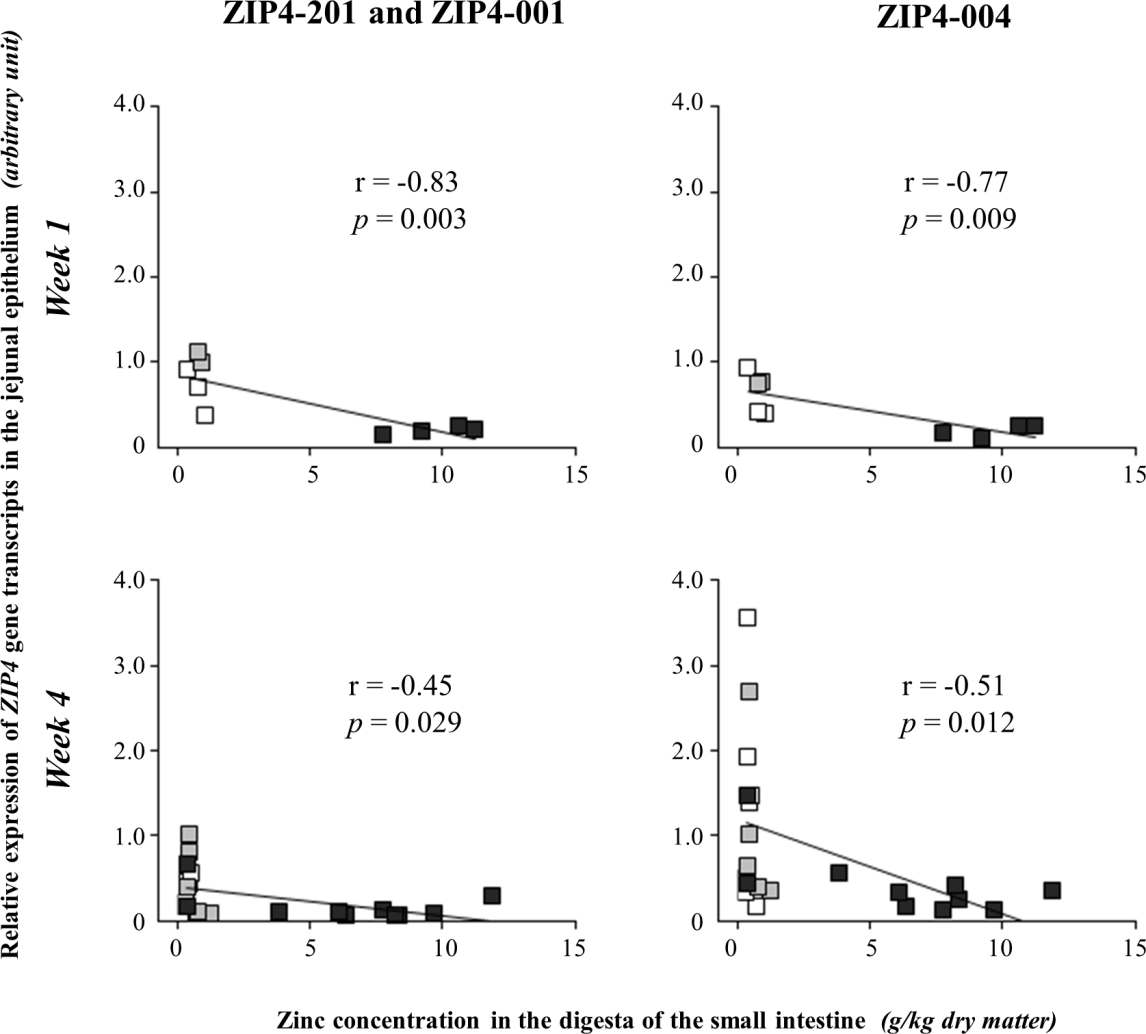
Correlation of zinc concentration in jejunal digesta with respective *ZIP4* expression in jejunal epithelium.

To assess the effect of the local zinc concentration in the lumen of the small intestine on the expression of *ZIP4* transcripts in the lumenal tissue, Spearman’s correlation coefficients were calculated between individual zinc concentrations in the digesta of the small intestine and the *ZIP4* transcript amounts in the jejunal epithelium of piglets on the different diets for one and four weeks. The analysis provided evidence for a negative correlation of higher zinc concentrations in the digesta with lower expression of *ZIP4* transcripts (Fig 2). Across all zinc diets, this relationship was stronger after one week (ZIP4-201 and ZIP4-001: r = -0.83, *p* = 0.003; ZIP4-004: r = -0.77, *p* = 0.009) than after four weeks feeding (ZIP4-201 and ZIP4-001: r = -0.45, *p* = 0.029; ZIP4-004: r = -0.51, *p* = 0.012). The piglets of the HZn group had the highest zinc concentrations in the digesta and showed the lowest expression of *ZIP4* after one as well as after four weeks.

Shown are the Spearman’s correlation coefficients for the relationship between individual zinc concentrations in the digesta of the small intestine with respective relative gene expression of the *ZIP4* gene transcripts ZIP4-201, ZIP4-001, and ZIP4-004 in the jejunal epithelium for one and four weeks of feeding different zinc diets. Individuals of different zinc diet groups are labelled as follows: white square: LZn (low dietary zinc) = 57 mg zinc/kg feed, n _Week1_ = 3, n _Week4_ = 6; grey square: NZn (normal dietary zinc) = 164 mg zinc/kg feed, n _Week1_ = 2, n _Week4_ = 7; black square: HZn (high dietary zinc) = 2,425 mg zinc/kg feed, n _Week1_ = 5, n _Week4_ = 10.

### The effects of dietary zinc concentrations and the length of the feeding period on the methylation status of the *ZIP4* Gene

**Fig 3:**
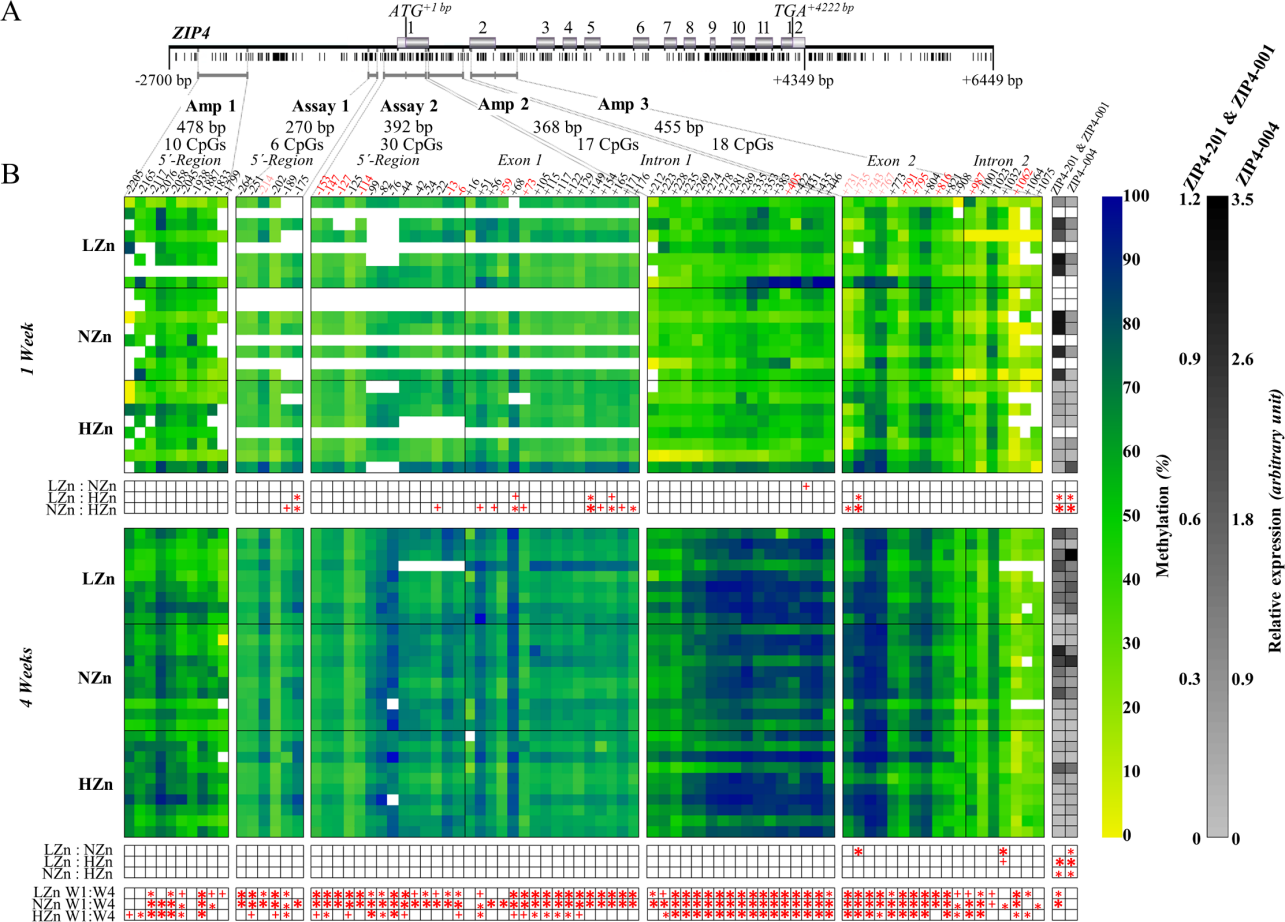
Methylation status of *ZIP4* gene regions and expression in jejunal epithelium after different zinc treatments.

The analysis of the methylation status of putative regulatory regions of the *ZIP4* gene in the jejunal epithelium provided evidence for an epigenetic regulation of the *ZIP4* gene. Feeding the three different zinc diets (LZn, NZn, and HZn) for four weeks led to a comprehensive increase of methylation of CpG sites in a region of the *ZIP4* gene comprising the examined upstream region and the first two exons and introns compared to one week feeding (3.7·10^-10^ < *p* < 0.099) (Fig 3, S2 Table). The change was less pronounced in animals of the HZn diet group compared to all others.

Shown is the methylation status of CpGs in putative regulatory regions of the *ZIP4* gene and the respective relative gene expression of the *ZIP4* gene transcripts in the jejunal epithelium of the small intestine of piglets fed with different zinc concentrations for one and four weeks. (A) Structure of the porcine *ZIP4* gene, distribution of CpGs (black dashes), and location of analysed regions. (B) Relative methylation status of CpGs of sequenced *ZIP4* fragments and the relative expression of ZIP4-201, ZIP4-001, and ZIP4-004 transcripts in different feeding groups after one and four weeks of feeding. Abbreviations: LZn (low dietary zinc) = 57 mg zinc/kg feed, methylation: n _Week1_ = 8, n _Week4_ = 9, expression: n _Week1_ = 5, n _Week4_ = 9; NZn (normal dietary zinc) = 164 mg zinc/kg feed, methylation: n _Week1_ = 8, n _Week4_ = 10, expression: n _Week1_ = 4, n _Week4_ = 10; HZn (high dietary zinc) = 2,425 mg zinc/kg feed, methylation: n _Week1_ = 8, n _Week4_ = 10, expression: n _Week1_ = 7, n _Week4_ = 10. The yellow-to-blue colour coded matrix shows the relative methylation status of the analysed CpGs of the amplified fragments. Columns: Every column represents a CpG at a specific position in the *ZIP4* gene relative to the TSS (ATG). CpGs in amplicons were examined by Sanger sequencing of DNA fragments after PCR of bisulfite-treated whole genome DNA. CpGs in assays were examined by pyrosequencing of DNA fragments after PCR of bisulfite-treated whole genome DNA. Lines: Every line represents an individual animal of the different zinc feeding groups. Colours: The yellow-to-blue colour coded matrix shows the methylation status with yellow = 0% and blue = 100% methylation. White blocks within the colour matrix represent missing methylation data. The grey-to-black colour coded matrix shows the relative expression of the *ZIP4* transcripts with light grey = no relative difference and black = 1.2-fold and 3.5-fold relative transcript amount difference for ZIP4-201 & ZIP4-001 and ZIP4-004, respectively. White cells are missing data. Expression values are given as relative values to the mean of the ΔCt values of the NZn group after one week NZn diet feeding. Columns: The first column represents ZIP4-201 and ZIP4-001 (long transcripts, differing in the TSS) and the second column ZIP4-004 (short transcript). Lines: Every line represents an individual animal of the different zinc feeding groups. Both matrices are divided into an upper and a lower part for one and four weeks of feeding different zinc concentrations, respectively. Bonferroni corrected Student’s t-test was performed for multiple testing for differences of the methylation degree at a particular CpG or of the expression for a particular transcript among the zinc feeding groups after one and four weeks of feeding. Bilateral Student’s t-test was performed for comparison of the methylation at a particular CpG or of the expression for a particular transcript between one and four weeks of feeding within the same feeding group. * = *p* < 0.01, * = *p* < 0.05, + = *p* < 0.1. Red coloured CpGs above the yellow-to-blue colour coded matrix indicate negative correlation between their methylation percentage and the expression of the transcripts ZIP4-201 and ZIP4-001; rose coloured CpGs indicate negative correlation between their methylation percentage and the expression of the transcript ZIP4-004.

After one week, feeding of the HZn diet was correlated with a higher methylation of three CpGs in the 5´-region, nine CpGs in exon 1, and two CpGs in exon 2 by 13% up to 90% compared to the LZn and NZn diets (8.0·10^-4^ < *p* < 0.105) (Fig 3, S2 Table). It is conspicuous that we observed this higher difference of methylation between the feeding groups at CpGs located in the second exon than in the 5´-regulative region, exon 1 or intron 1. Four weeks feeding of the NZn diet led to higher methylation at CpG +735 in exon 2 (*p* = 0.008) and CpG +1032 in intron 2 (*p* = 0.007) by 16% and 28%, respectively, when compared to the LZn diet group (Fig 3, S2 Table).

The methylation status of some CpGs likely had an influence on the expression of the long transcripts ZIP4-201, ZIP4-001 and the short transcript ZIP4-004. The increased methylation of seven CpGs in the 5´-region (CpGs -214, -153, -147, -127, -114, -13, -6), two CpGs in exon 1 (CpGs +59, +73), CpG +405 in intron 1, five CpGs in exon 2 (CpGs +735, +767, +791, +795, +816), and two CpGs in intron 2 (CpGs +987, +1062) were significantly negatively associated with the expression of the long transcripts ZIP4-201 and ZIP4-001 (8.0·10^-4^ < *p* < 0.050) (Fig 3, S3 Table). Furthermore, a higher methylation level at CpG +743 in the beginning of exon 2 was also negatively associated with the expression of the ZIP4-004 transcript (0.005 < *p* < 0.020) (Fig 3, S4 Table). In addition to the action of single CpGs, also the combination of CpGs had significant effects on the transcript amounts into the same direction as single CpGs (e.g. CpGs in the 5´-region -147 and -127, -13 and -6, S3 Table).

We like to emphasise that the methylation of CpG +731 and CpG +735 at the beginning of exon 2 was highly significant (8.3·10^-4^ < *p* < 0.028) affected by the dietary zinc concentration both after one and four weeks of feeding different zinc diets (S2 Table). The higher methylation values for these CpGs were strongly negatively associated with the expression of *ZIP4*, which is consistent with our expectation of the direction of effect on the transcription (Fig 3, S3 and S4 Tables).

The relationship between the methylation and expression of the *ZIP4* transcripts ZIP4-201 and ZIP4-001 across all zinc diet groups was higher after one week than after four weeks of feeding (S2 Fig, S3 Table). For instance, the correlation coefficient between methylation at CpG +735 in exon 2 and expression of the long *ZIP4* transcripts was r = -0.65 (*p* < 0.007) after one week, whereas this correlation disappeared until four weeks (r = 0.04, *p* = 0.843). The correlation between CpG methylation and expression of ZIP4-201 and ZIP4-001 also decreased with increasing zinc concentration in the diet (e.g. CpG -153 in the 5´-region after one week of feeding: LZn r = -0.90, *p* = 0.037; NZn r = -0.80, *p* = 0.200; HZn r = -0.07, *p* = 0.879) (S2 Fig).

**Fig 4:**
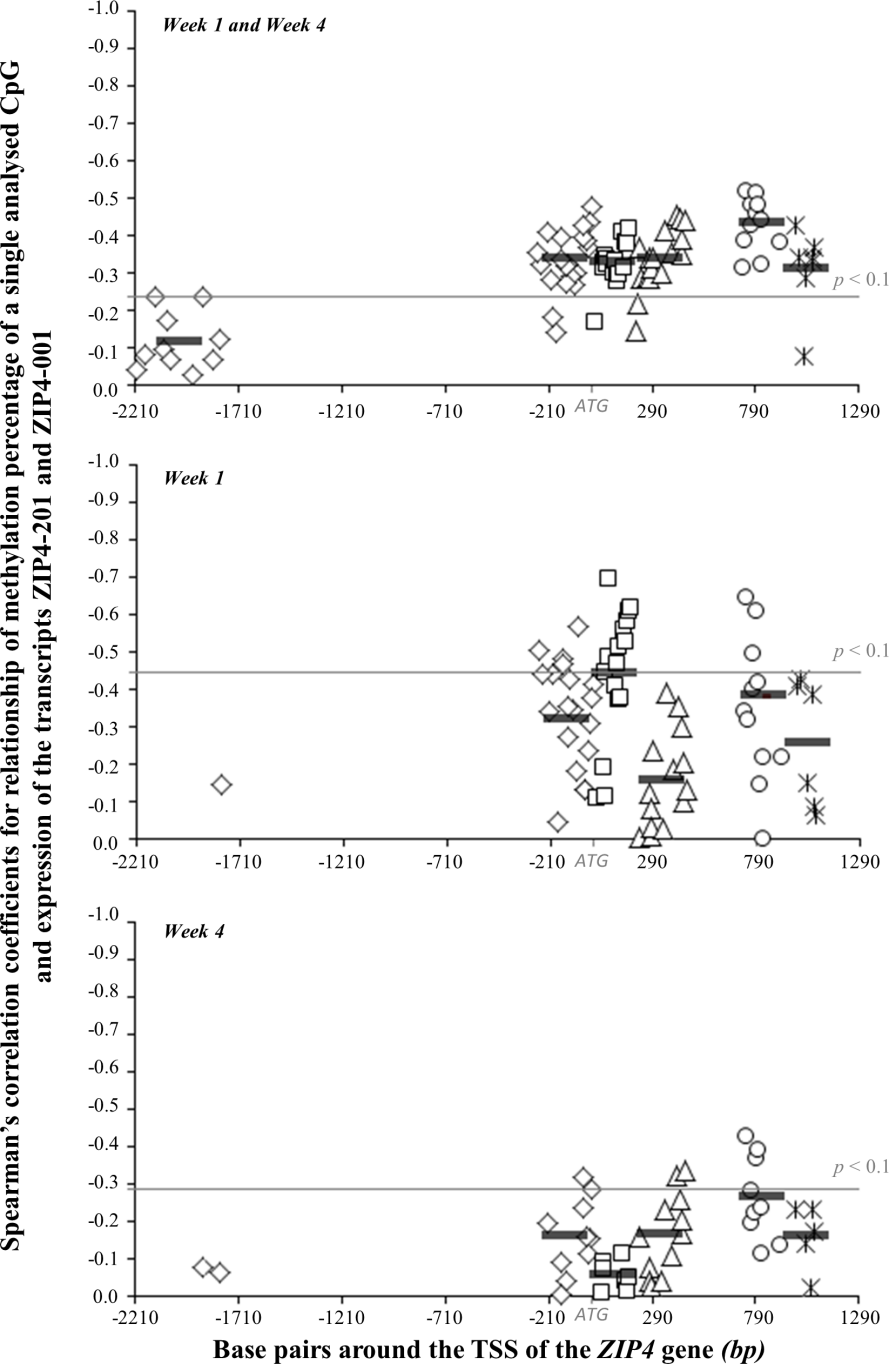
Influence of methylation of *ZIP4* gene regions on expression of long transcripts in jejunal epithelium.

Considering the correlation between the methylation level of all analysed CpGs in the *ZIP4* gene and expression of the long transcripts across all feeding groups and both feeding periods (week 1+4), we found that the CpGs in exon 2 had the highest influence on transcription (Fig 4). In this region, the average correlation coefficient between the methylation of all analysed CpGs in all animals and the expression of the long transcripts was r_Average_ = -0.43 (2·10^-4^ < *p* < 0.036). The influence of the methylation of the CpGs in other regions around the TSS, exon 1, intron 1, and intron 2 were similar and below -0.34. (5´-region: r_Average_ = -0.34, 0.001 < *p* < 0.371; exon 1: r_Average_ = -0.33, 0.004 < *p* < 0.271; intron 1: r_Average_ = -0.34, 0.002 < *p* < 0.357; intron 2: -0.31, 0.003 < *p* < 0.617). CpGs in the region around 2000 bp upstream of the TSS did not significantly contribute to the regulation of the *ZIP4* gene. The comparison of the one and four weeks feeding periods showed once more a higher relationship between methylation and transcription after one week than after four weeks. After one week, the CpGs in exon 1 showed the highest influence on the expression of ZIP4-201 and ZIP4-001 (r_Average_ = -0.44, 0.006 < *p* < 0.703) whereas three CpGs in exon 2 had the highest influence after a feeding period of four weeks (CpG +743: r = -0.43, *p* = 0.021; CpG +795: r = -0.37, *p* = 0.050; CpG +804: r = -0.39, *p* = 0.035). The influence of the CpGs in exon 1 on the transcription after one week of feeding the different zinc diets disappeared after four weeks.

Plotted are the negative Spearman’s correlation coefficients for the relationship of the methylation percentage of all analysed CpGs and the expression of the transcripts ZIP4-201 and ZIP4-001 in the jejunal epithelium of the small intestine of piglets fed zinc for one and four weeks over all diet groups at the y-axis against the location of the CpGs around the TSS of the *ZIP4* gene. The correlation coefficients for the 5´-region of the *ZIP4* gene are split in two sections since we analysed a region around 2000 bp before the TSS and a region near the TSS. Correlation coefficients of different regulative regions of *ZIP4* are labelled as follows: rhombus: 5´-regulative region (30 CpGs), square: exon 1 (16 CpGs), triangle: intron 1 (17 CpGs), circle: exon 2 (11 CpGs), star: intron 2 (7 CpGs). Per CpG: n _Week1_ = 7-16, n _Week4_ = 25-29, n _Week1+4_ = 35-45. Abbreviations: TSS = transcription start site (ATG). *p*-value cut off line separates significant correlation coefficients or values by trend above the line from non-significant coefficients below the line.

### Identifying potential transcription factor binding sites at differentially methylated CpGs

Thirteen transcription factors were identified *in silico*, whose core binding sites overlap with the analysed CpG positions (S5 Table). The differentially methylated CpG -189 and CpG -175 in the 5´-region and CpG +154, CpG +165, CpG +171 and CpG +176 in exon 1 overlap with potential binding sites for the B-cell lineage specific activator protein (BSAP), the E2F transcription factor 1 (E2F), the transcriptional enhancer factor 1 (TEF-1), the aryl hydrocarbon receptor nuclear translocator (ARNT), the neuroblastoma MYC oncogene (n-MYC), the upstream transcription factor 1 (USF), the cAMP responsive element binding protein 1 (CREB), the complex named heart and neural crest derivatives expressed 1 (HAND1), and the transcription factor 3 (E47). BSAP may play an important role in B-cell differentiation as well as neural development and spermatogenesis, E2F regulates cell cycle and DNA replication, TEF-1 is involved in organ size control and tumour suppression, ARNT is required for activity of the dioxin receptor and activates genes involved in metabolism, angiogenesis and apoptosis, n-MYC is associated with a variety of neuroblastoma tumours and activates the transcription of growth-related genes, USF is an ubiquitous transcription factor that binds to a variety of viral and cellular promoters, CREB stimulates transcription upon binding to the DNA cAMP response element and is involved in different cellular processes including the synchronization of circadian rhythmicity or the differentiation of adipose cells, HAND1 plays an essential role in cardiac morphogenesis, and E47 is involved in the initiation of neuronal differentiation. The binding sites of seven of these nine transcription factors (BSAP, ARNT, n-MYC, USF, CREB, E47, and HAND1) and of the transcription factors proto-oncogene c-Rel (c-Rel) and Myc-associated factor x (Max) are located in intron 1 and exon 2, whose CpGs where extremely higher methylated after four weeks of feeding the different diets. c-Rel may play a role in differentiation and lymphopoiesis whereas Max forms a sequence-specific DNA-binding protein complex with the transcription factors Myc or Mad and acts as transcriptional regulator. Most of all the above mentioned transcription factors can act as transcriptional activators. In turn, methylation of their DNA binding sites would impair transcription.

## Discussion

We examined the methylation status of the *ZIP4* gene in weaning piglets fed diets with different amounts of zinc oxide. The most significant finding of the study is the increased methylation of almost all CpG sites in the analysed putative regulatory regions after feeding different zinc concentrations for four weeks. Furthermore, minor methylation differences were found in response to the diets after one and four weeks. These minor differences were higher in CpGs located in the second exon than in the 5´-regulatory region, intron 1 and exon 1. Moreover, after one week, one CpG site in the second exon (CpG +735) was on average 56% higher methylated on the HZn diet when compared to the other diet groups; this CpG was also associated with lower expression of the long *ZIP4* transcripts.

Our data provide evidence for local zinc concentrations in the small intestine that mirror the different zinc concentrations in the diets. In addition, we could confirm a strong negative correlation between the zinc concentration in the diet and the expression of *ZIP4* transcripts in the jejunal epithelium [18,19,38–42]. After a feeding period of four weeks, the expression of the long *ZIP4* transcripts was about the same in the LZn and the NZn groups, while the short transcript was differently regulated with higher transcript amounts in the LZn group. This finding suggests that the expression of the short transcript is more sensitive to the available zinc concentration. This sensitivity might be influenced by higher RNA stability of the short transcript, since a negative correlation between mRNA length and stability was found in humans on a genome-wide scale [43]. Our data are supported by a feeding experiment with mice together with cell culture experiments, where an influence of zinc on the mRNA stability of the *ZIP4* gene was shown [42,44]. These experiments indicated that zinc may either induce a downregulation of a protein that is involved in maintaining stability of *ZIP4* mRNA or destabilize *ZIP4* mRNA.

Since changes in the methylation of the single CpG +743 in the second exon affected the expression of the short transcript but not of the long transcripts, we assume that the short transcript might be regulated by the region in exon 2, where CpG +743 is located. However, because this region is far away from the transcription start site of the ZIP4-004 transcript, which is probably located in intron nine, it remains unclear, if the methylation of CpGs in intron nine, which we did not analyse, plays a bigger role in the regulation of ZIP-004 than the methylation of the CpGs we analysed in this study.

Although we could provide evidence for the existence of the short ZIP4-004 transcript using exon-specific RT-qPCR (data not shown), the question arises whether the short protein product would be able to transport zinc ions at all. Structure prediction analyses revealed that the protein of the long transcript ZIP4-201 consists of eight transmembrane domains with four cytoplasmic and three extracellular inter-transmembrane loops as well as an extracellular N-terminal and C-terminal end of the protein. The short transcript contains only the last five transmembrane helices with only two cytoplasmic and two extracellular loops whereas the N-terminal end of the protein product is predicted to be located intracellular. The simplified structure of the protein product of ZIP4-004 implies at least insufficient transport of zinc ions. Furthermore, a zinc-uptake assay following expression of hZIP4 in *Xenopus laevis* oocytes indicated that extracellularly accessible histidine residues of hZIP4, which are predominantly found at the N-terminal protein end, are required for cation uptake [45]. Since the N-terminal end of the porcine protein of ZIP4-004 is located intracellular and has only one histidine residue we suggest that the ZIP4-004 protein might be severely impaired or even inoperable for the transport of zinc ions.

In part the transcriptional changes of the *ZIP4* transcripts could be linked to methylation changes in the examined *ZIP4* gene regions. The drastic increase of the methylation of almost all CpGs in the analysed putative regulatory regions of the *ZIP4* gene after four weeks feeding the same diet was observed independently of the dietary zinc concentration. These regions do not overlap with repetitive elements, which are prone to be silenced. We assume that even the LZn and NZn groups received a dietary zinc level, which slightly exceeded the requirements, thus leading to a cellular response to prevent a zinc overload by increased methylation patterns. Feeding recommendations for optimal zinc supply for weaning piglets (i.e. 100 mg zinc/kg feed) take into account safety margins due to low bioavailability of inorganic zinc sources and interaction with dietary factors such as phytate and calcium [14,15]. However, recent findings indicate that dietary zinc levels between 50 to 60 mg zinc/kg might already be sufficient for healthy growth [46]. Therefore, down-regulation of the expression of the ZIP4-201 and ZIP4-001 transcripts after four weeks indicates a regulatory mechanism to maintain zinc homeostasis even in the LZn and NZn diet groups. Recently, it was shown that high zinc dosages are beneficial only during the early phase of the post weaning period [19]. During this time, the body takes up higher zinc amounts necessary to facilitate all bodily functions for the fastest growth phase. This is also consistent with our observation that the *ZIP4* gene may be open for direct zinc effects and high transcription rates while the high methylation pattern found after a feeding period longer than one week prevents the uptake of high zinc through decrease of zinc transporter expression.

Since we cannot distinguish between the influences of age of piglets, length of feeding period and other effects on the observed switch of methylation pattern found between one and four weeks of feeding, additional processes that take place in the gut have to be considered to affect epigenetic processes. For example, zinc as an essential micronutrient, could directly affect the growth of microbiota and change the composition of gut microbiota [13,47]. Since microbiota produce short chain fatty acids, such as butyrate, which are well known as histone deacetylase inhibitors that stimulate histone acetylation, they may contribute to modify DNA methylation through relaxed euchromatin accessible for DNA methyltransferases [48,49].

The regulation of the zinc absorption and the body zinc balance seem to be primary determined by the intestinal uptake efficiency [50]. The effect of ageing on the intestinal zinc absorption is poorly understood and may be related to different physiological requirements which change with age, which in turn is associated with changes in lean body mass, energy requirements, and physical activity [51]. Many human studies showed a gradual decline of zinc intake with age, but it is not exactly known how this phenomenon impacts or is influenced by the regulation of zinc transporters. Two independent cell culture experiments showed a dysregulation of the expression of the zinc transporter genes *Zip6* and *ZnT5* with ageing which might be correlated with increased promotor methylation of these genes [52,53]. We analysed the expression and methylation of the *ZIP4* gene of piglets during the early age from weaning to youth, which is a phase of highest growth rate with a high demand of metabolic energy and nutrients of all kind. The length of the zinc feeding period plays a considerable role, since the feeding of the HZn diet was only beneficial until the end of week two whereas afterwards detrimental effects were seen [19]. Our finding is further supported by feeding studies with weaning pigs which received diets with 4,000 to 8,000 kg zinc/kg feed, where the high zinc concentrations also showed toxic effects leading to depressed growth and appetite as well as increased mortality rates [54].

With respect to minor changes of methylation of single CpGs among the different zinc diets, our data provide evidence for an interaction of the zinc concentration in the feed and the methylation at CpG +735 in exon 2. Both factors had an influence on the expression of the *ZIP4* transcripts. A higher zinc supply and a higher methylation status are associated with lower expression of the *ZIP4* transcripts, a direction of effects we had expected. For the zinc transporter *ZIP8* it has already been shown that down-regulation of the gene expression was caused, at least partly, by hypermethylation of a CpG island in the promotor of the gene [55]. The increasing methylation change with increasing distance from the TSS might show that the CpGs, which are located further down-stream of the TSS, are more important for the transcriptional regulation of the *ZIP4* gene to improve the efficiency of the transcriptional process by impairing unprofitable transcriptional initiation within transcribed regions [56].

Since the higher methylation values at the end of the longer feeding period was independent from the fed diet, we suggest that the methylation likely serves as an adaptive mechanism for the regulation of *ZIP4* gene expression to maintain zinc homeostasis in the long term. In contrast, the low *ZIP4* expression in animals of the HZn group seen after one week might be driven by zinc-responsive transcription factors. Such short-term gene regulation that acts fast to maintain a necessary homeostasis has been shown, for example, for the metallothionein gene and its metal response element binding transcription factor 1 [57,58].

The correlation analysis between methylation status of analysed CpGs and *ZIP4* gene expression indicated that the methylation of single CpGs rather plays a role after one week than after four weeks of feeding, where nearly all CpGs were higher methylated. After one week of feeding, the expression of the *ZIP4* gene transcripts seems to be more regulated by differential methylation of single CpGs under physiological zinc concentrations in the range of the LZn and NZn diets compared to pharmacological high amounts in the HZn diet. Besides the meaning in possible long-term regulation, DNA-methylation of single CpGs in the *ZIP4* gene is therefore also suggested to be a fine tuning mechanism for the regulation of the transcriptional expression under certain dietary conditions, while other mechanisms like zinc-responsive transcription factors may rather lead to a significant down-regulation of the *ZIP4* transcription on very high zinc concentrations.

Transcription factor binding site analysis revealed the potential binding of nine transcription factors in the first intron and second exon, but none of the binding sites of these transcription factors include the CpG +735 in exon 2, which turned out to be the most prominent CpG. The two transcription factors CREB and BSAP that potentially bind to differentially methylated CpGs are zinc finger proteins that bind a zinc ion which is important for both structure and function of the proteins [59–61]. CREB was shown to respond to higher zinc amounts triggered by an overexpression of *ZIP4* or other zinc transporters in several cell systems [60–64] and evidence exist that CREB binds to unmethylated cAMP response elements [65]. Therefore, the higher methylation of the *ZIP4* gene after four weeks and the lowered intake of zinc ions through a down-regulated expression of the *ZIP4* transcripts might hinder the CREB transcription factor in binding to the DNA. Furthermore, for the transcription factor USF it was also shown that CpG methylation at the USF-binding site inhibits the binding of USF [66–68]. The binding site of Krüppel-like factor 4, a transcription factor for which it was shown to regulate *Zip4* expression in mouse small intestine [69], did not overlap with any of the analysed CpG positions.

In future experiments it will be necessary to identify the optimal dosage, time point, and duration of feeding zinc as a feed additive to understand the conditions under which the major methylation changes occur and if they are reversible. Additional investigations have to be performed to assess the effect of the different zinc concentrations in the feed on the health of piglets. The weight gain of piglets in the present study has been reported previously [19]. The growth curves showed a significantly higher body weight in the first two weeks in the HZn group compared to the other groups. Afterwards, the body weights in the NZn and LZn groups became higher indicating a negative effect of pharmacological zinc concentrations in the HZn group when applied longer than two weeks.

## Conclusions

The data of this study provide evidence for DNA methylation as a mechanism for the regulation of the expression of the *ZIP4* transcripts in the jejunal epithelium of growing weaned piglets. We could show that the zinc concentration in the feed and the length of the feeding period and/or the age had influence on the methylation pattern. The major changes in the methylation pattern between one and four weeks of feeding different diets containing normal, low, and very high zinc concentrations led us to the conclusion that massive methylation in putative regulatory regions of the *ZIP4* gene might be a mechanism of gene regulation in the long term and probably does not play a role in acute short-term response to high dietary zinc concentrations. Since there was only one CpG across all diet groups that was influenced by the dietary zinc concentration and that had also an effect on the *ZIP4* expression, we conclude that DNA methylation in response to the dietary zinc concentration may have only minor regulatory effects. DNA methylation of single CpGs could serve as a fine tuning mechanism for *ZIP4* gene regulation.

## Supporting Information

S1 Fig Porcine *ZIP4* gene and its known protein coding transcripts. (A) Scheme of the structure of the porcine *ZIP4* gene and distribution of CpGs (black dashes). (B) Protein coding transcripts of the porcine *ZIP4* gene. The transcripts ZIP4-201 and ZIP4-001 contain all 12 exons of the *ZIP4* gene. ZIP4-001 has a shortened exon 1 with an aback shifted TSS. The transcript ZIP4-003 includes the last five exons of the *ZIP4* gene with a prolonged exon 8 towards the TSS. The transcript ZIP4-004 contains the last three exons 10 to 12 with an elongated exon 10 beginning at the end of intron 9. In this paper, only the transcripts ZIP4-201, ZIP4-001 and ZIP4-004 were subjects of matter. Both, the long transcripts ZIP4-201 and ZIP4-001 were undistinguishable during transcript expression analysis and were measured together with the same primer pair.
(TIF)

S2 Fig Correlation of exclusive *ZIP4* CpG methylation with the expression of long transcripts in jejunal epithelium. Shown is the Spearman’s correlation between the methylation of exclusive single CpGs or group of CpGs and the expression of the *ZIP4* transcripts ZIP4-201 and ZIP4-001 in the jejunal epithelium of the small intestine of piglets fed different zinc concentrations for one and four weeks. For every shown CpG or group of CpGs the expression of the transcripts ZIP4-201 and ZIP4-001 at the y-axis is plotted against the methylation percentage of the CpGs at the x-axis. Individual coefficient values of the different zinc diet groups are labelled as follows: white square: LZn (low dietary zinc) = 57 mg zinc/kg feed, n _Week1_ = 5, n _Week4_ = 9; grey square: NZn (normal dietary zinc) = 164 mg zinc/kg feed, n _Week1_ = 4, n _Week4_ = 10; black square: HZn (high dietary zinc) = 2,425 mg zinc/kg feed, n _Week1_ = 7, n _Week4_ = 10. Correlation coefficients (r) and respective *p*-values of every diet group or over all diet groups (dashed square) are shown in every graph. Abbreviations: 5´-Reg = 5´-regulative region.
(TIF)

S1 Table Influence of zinc on *ZIP4* expression in jejunal epithelium after one and four weeks treatment. Shown is the influence of dietary zinc concentrations on relative expression of the *ZIP4* gene transcripts in the jejunal epithelium of the small intestine of piglets after one and four weeks of feeding. Abbreviations: LZn, low dietary zinc = 57 mg zinc/kg feed; NZn, normal dietary zinc = 164 mg zinc/kg feed; HZn, high dietary zinc = 2,425 mg zinc/kg feed. All expression values of the transcripts are relative to the mean of the ΔCt values of the NZn group after one week NZn diet feeding. ^a,b,c^Labelled means with different letters in a row are significantly different (*p* < 0.05). ^A,B^Labelled means with different letters in a column are significantly different (*p* < 0.05).
(DOCX)

S2 Table Influence of zinc on the *ZIP4* gene region methylation in the jejunal epithelium of piglets. Shown is the influence of dietary zinc concentrations on the methylation status of CpGs in putative regulatory regions of the *ZIP4* gene in the epithelium of the jejunal intestine of piglets fed the different zinc diets. Abbreviations: LZn, low dietary zinc = 57 mg zinc/kg feed, n _Week1_ = 8, n _Week4_ = 9; NZn, normal dietary zinc = 164 mg zinc/kg feed, n _Week1_ = 8, n _Week4_ = 10; HZn, high dietary zinc = 2,425 mg zinc/kg feed, n _Week1_ = 8, n _Week4_ = 10. ^a,b^Labelled means with different letters in a row are significantly different (*p* < 0.05). ^*a*.*b*^Labelled means with different letters in a row are different by trend (*p* ≤ 0.1).
(DOCX)

S3 Table Correlation of *ZIP4* methylation with the expression of the long transcripts in the jejunal epithelium. Shown are the Spearman’s correlation coefficients between the methylation of CpGs of the *ZIP4* gene and expression of the transcripts ZIP4-201 and ZIP4-001 over all diet groups for feeding periods of one (1) or four (4) weeks or both periods together (1+4) in the jejunal epithelium of the small intestine of piglets fed the different zinc diets.
(DOCX)

S4 Table Correlation of *ZIP4* methylation with the expression of the short transcript in the jejunal epithelium. Shown are the Spearman’s correlation coefficients between the methylation of CpGs of the *ZIP4* gene and expression of the transcript ZIP4-004 over all diet groups for feeding periods of one (1) or four (4) weeks or both periods together (1+4) in the jejunal epithelium of the small intestine of piglets fed the different zinc diets.
(DOCX)

S5 Table Transcription factors which binding sites overlap with analysed CpG positions. (DOCX)
