# Supplementary figures and images for: The Zinc Concentration in the Diet and the Length of the Feeding Period Affect the Methylation Status of the ZIP4 Zinc Transporter Gene in Piglets
Source: PLoS One. 2015 Nov 23;10(11):e0143098. doi: 10.1371/journal.pone.0143098 (PMC4658085; doi:10.1371/journal.pone.0143098)

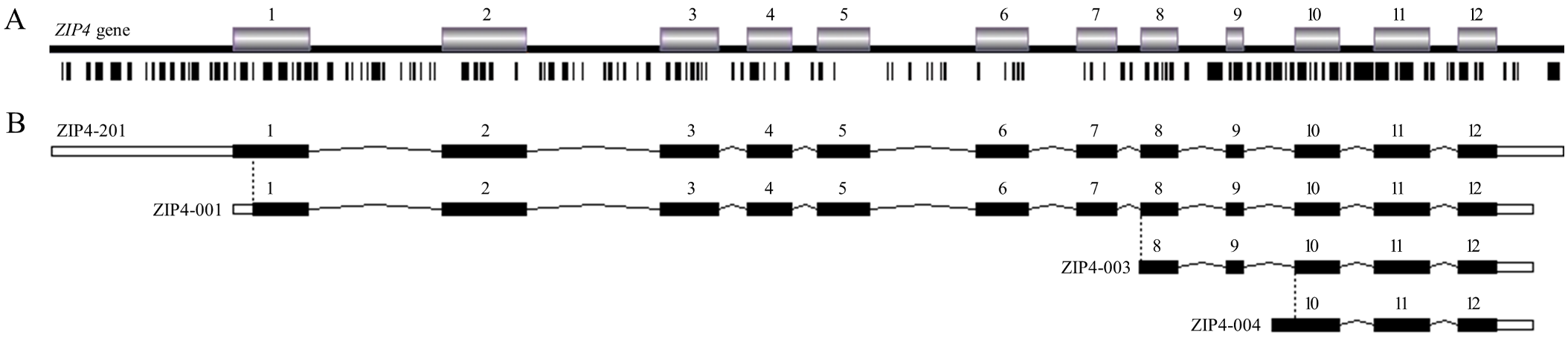

Supplement: S1 Fig — (A) Scheme of the structure of the porcine ZIP4 gene and distribution of CpGs (black dashes). (B) Protein coding transcripts of the porcine ZIP4 gene. The transcripts ZIP4-201 and ZIP4-001 contain all 12 exons of the ZIP4 gene. ZIP4-001 has a shortened exon 1 with an aback shifted TSS. The transcript ZIP4-003 includes the last five exons of the ZIP4 gene with a prolonged exon 8 towards the TSS. The transcript ZIP4-004 contains the last three exons 10 to 12 with an elongated exon 10 beginning at the end of intron 9. In this paper, only the transcripts ZIP4-201, ZIP4-001 and ZIP4-004 were subjects of matter. Both, the long transcripts ZIP4-201 and ZIP4-001 were undistinguishable during transcript expression analysis and were measured together with the same primer pair. (TIF) [file pone.0143098.s001.tif]

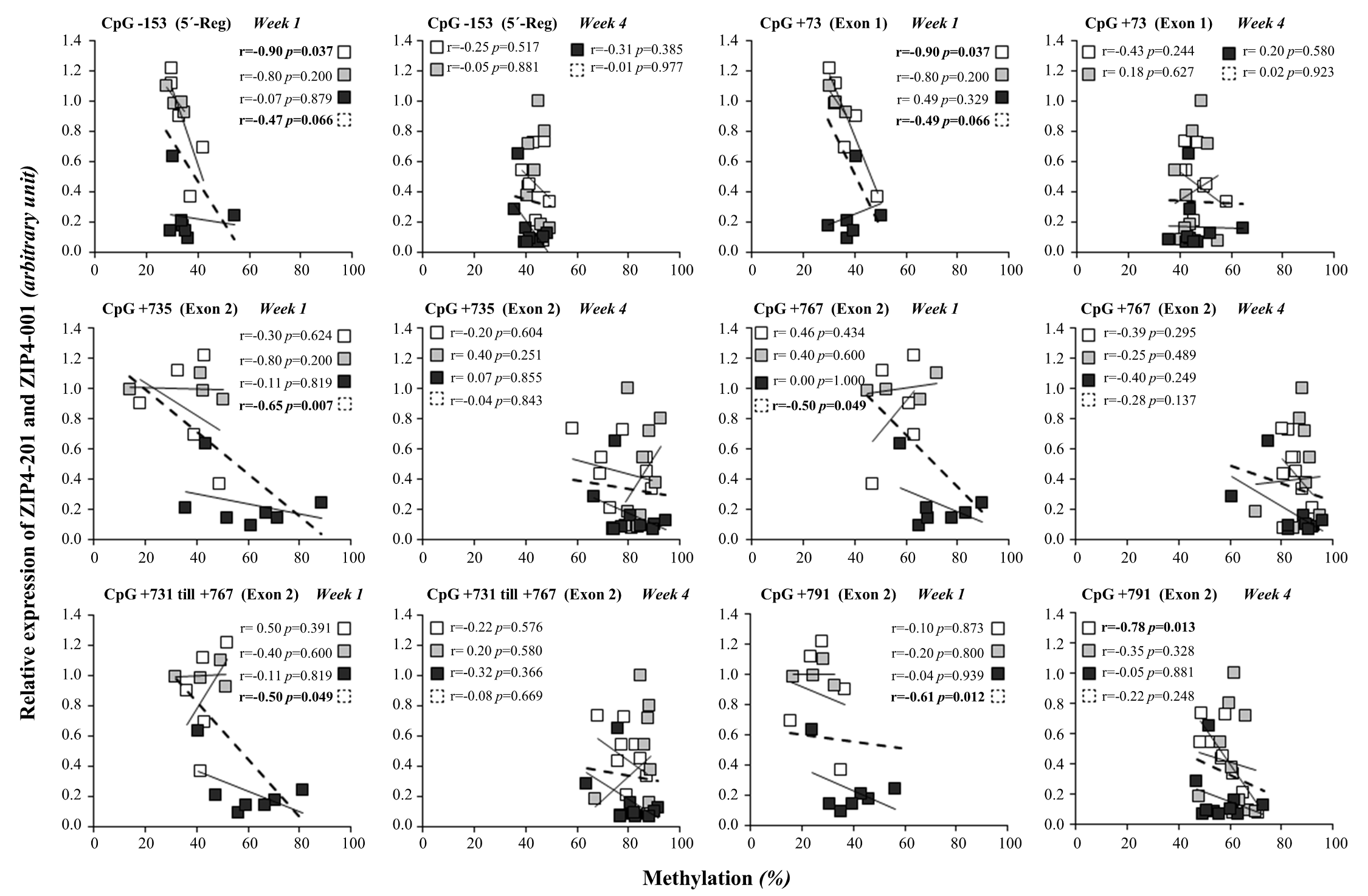

Supplement: S2 Fig — Shown is the Spearman’s correlation between the methylation of exclusive single CpGs or group of CpGs and the expression of the ZIP4 transcripts ZIP4-201 and ZIP4-001 in the jejunal epithelium of the small intestine of piglets fed different zinc concentrations for one and four weeks. For every shown CpG or group of CpGs the expression of the transcripts ZIP4-201 and ZIP4-001 at the y-axis is plotted against the methylation percentage of the CpGs at the x-axis. Individual coefficient values of the different zinc diet groups are labelled as follows: white square: LZn (low dietary zinc) = 57 mg zinc/kg feed, n Week1 = 5, n Week4 = 9; grey square: NZn (normal dietary zinc) = 164 mg zinc/kg feed, n Week1 = 4, n Week4 = 10; black square: HZn (high dietary zinc) = 2,425 mg zinc/kg feed, n Week1 = 7, n Week4 = 10. Correlation coefficients (r) and respective p-values of every diet group or over all diet groups (dashed square) are shown in every graph. Abbreviations: 5´-Reg = 5´-regulative region. (TIF) [file pone.0143098.s002.tif]
