# Supplementary material for: The Zinc Concentration in the Diet and the Length of the Feeding Period Affect the Methylation Status of the ZIP4 Zinc Transporter Gene in Piglets
Source: PLoS One. 2015 Nov 23;10(11):e0143098. doi: 10.1371/journal.pone.0143098 (PMC4658085; doi:10.1371/journal.pone.0143098)
Supplement: S1 Table — Shown is the influence of dietary zinc concentrations on relative expression of the ZIP4 gene transcripts in the jejunal epithelium of the small intestine of piglets after one and four weeks of feeding. Abbreviations: LZn, low dietary zinc = 57 mg zinc/kg feed; NZn, normal dietary zinc = 164 mg zinc/kg feed; HZn, high dietary zinc = 2,425 mg zinc/kg feed. All expression values of the transcripts are relative to the mean of the ΔCt values of the NZn group after one week NZn diet feeding. a,b,cLabelled means with different letters in a row are significantly different (p < 0.05). A,BLabelled means with different letters in a column are significantly different (p < 0.05). (DOCX) [file pone.0143098.s003.docx]

**S1 Table. Influence of zinc on *ZIP4* expression in jejunal epithelium after one and four weeks treatment.**

| Transcript | Feeding period, *weeks* | Relative expression, *arbitrary unit* | | | | | | | | | ANOVA  *p-*value _diet_ |
| --- | --- | --- | --- | --- | --- | --- | --- | --- | --- | --- | --- |
|  |  | LZn | | | NZn | | | HZn | | |  |
|  |  | *mean* | *SD* | *n* | *mean* | *SD* | *n* | *mean* | *SD* | *n* |  |
| ZIP4-201 ZIP4-001 | 1 | 0.86^a,A^ | 0.34 | 5 | 1.00^a,A^ | 0.08 | 4 | 0.17^b^ | 0.05 | 7 | < 5.1·10^-5^ |
|  | 4 | 0.45^a,B^ | 0.22 | 9 | 0.40^a,B^ | 0.34 | 10 | 0.12^b^ | 0.07 | 10 | < 0.014 |
| ZIP4-004 | 1 | 0.62^a^ | 0.34 | 5 | 0.73^a^ | 0.02 | 4 | 0.18^b^ | 0.06 | 7 | < 0.005 |
|  | 4 | 1.19^a^ | 0.63 | 9 | 0.63^b^ | 0.32 | 10 | 0.30^c^ | 0.15 | 10 | 6.2·10^-4^ |

Shown is the influence of dietary zinc concentrations on relative expression of the *ZIP4* gene transcripts in the jejunal epithelium of the small intestine of piglets after one and four weeks of feeding. Abbreviations: LZn, low dietary zinc = 57 mg zinc/kg feed; NZn, normal dietary zinc = 164 mg zinc/kg feed; HZn, high dietary zinc = 2,425 mg zinc/kg feed. All expression values of the transcripts are relative to the mean of the ΔCt values of the NZn group after one week NZn diet feeding. ^a,b,c^Labelled means with different letters in a row are significantly different (*p* < 0.05). ^A,B^Labelled means with different letters in a column are significantly different (*p* < 0.05).
