# Supplementary material for: The Zinc Concentration in the Diet and the Length of the Feeding Period Affect the Methylation Status of the ZIP4 Zinc Transporter Gene in Piglets
Source: PLoS One. 2015 Nov 23;10(11):e0143098. doi: 10.1371/journal.pone.0143098 (PMC4658085; doi:10.1371/journal.pone.0143098)
Supplement: S2 Table — Shown is the influence of dietary zinc concentrations on the methylation status of CpGs in putative regulatory regions of the ZIP4 gene in the epithelium of the jejunal intestine of piglets fed the different zinc diets. Abbreviations: LZn, low dietary zinc = 57 mg zinc/kg feed, n Week1 = 8, n Week4 = 9; NZn, normal dietary zinc = 164 mg zinc/kg feed, n Week1 = 8, n Week4 = 10; HZn, high dietary zinc = 2,425 mg zinc/kg feed, n Week1 = 8, n Week4 = 10. a,bLabelled means with different letters in a row are significantly different (p < 0.05). a.bLabelled means with different letters in a row are different by trend (p ≤ 0.1). (DOCX) [file pone.0143098.s004.docx]

S2 Table. Influence of zinc on the *ZIP4* gene region methylation in the jejunal epithelium of piglets.

| CpG position | *ZIP4* gene region | Feeding period, *weeks* | Relative methylation, *%* | | | | | | ANOVA  *p*-value _diet_ |
| --- | --- | --- | --- | --- | --- | --- | --- | --- | --- |
|  |  |  | LZn | | NZn | | HZn | |  |
|  |  |  | *LSMean* | *SE* | *LSMean* | *SE* | *LSMean* | *SE* |  |
| -189 | 5´-Region | 1 | 0.41*^ab^* | 0.04 | 0.35*^a^* | 0.04 | 0.46*^b^* | 0.04 | **< 0.040** |
| -175 | 5´-Region | 1 | 0.47^a^ | 0.04 | 0.41^a^ | 0.04 | 0.53^b^ | 0.04 | **< 0.014** |
| -22 | 5´-Region | 1 | 0.41*^ab^* | 0.03 | 0.31*^a^* | 0.04 | 0.43*^b^* | 0.03 | < 0.075 |
| +51 | Exon 1 | 1 | 0.58*^ab^* | 0.04 | 0.43*^a^* | 0.04 | 0.60*^b^* | 0.03 | **0.051** |
| +56 | Exon 1 | 1 | 0.47*^ab^* | 0.04 | 0.37*^a^* | 0.04 | 0.49*^b^* | 0.03 | < 0.063 |
| +68 | Exon 1 | 1 | 0.57^ab,^*^a^* | 0.03 | 0.53^a,^*^ab^* | 0.03 | 0.66^b,^*^b^* | 0.03 | **< 0.021** |
| +73 | Exon 1 | 1 | 0.37*^ab^* | 0.02 | 0.31*^a^* | 0.03 | 0.40*^b^* | 0.02 | 0.075 |
| +149 | Exon 1 | 1 | 0.43^a^ | 0.03 | 0.36^a^ | 0.03 | 0.52^b^ | 0.03 | **< 0.005** |
| +154 | Exon 1 | 1 | 0.41*^ab^* | 0.03 | 0.36*^a^* | 0.04 | 0.47*^b^* | 0.03 | < 0.085 |
| +165 | Exon 1 | 1 | 0.39^ab,^*^a^* | 0.03 | 0.34^a,^*^ab^* | 0.04 | 0.48^b,^*^b^* | 0.03 | **0.015** |
| +171 | Exon 1 | 1 | 0.44*^ab^* | 0.03 | 0.40*^a^* | 0.04 | 0.52*^b^* | 0.03 | 0.057 |
| +176 | Exon 1 | 1 | 0.37^ab^ | 0.02 | 0.33^a^ | 0.03 | 0.44^b^ | 0.02 | **< 0.025** |
| +431 | Intron 1 | 1 | 0.61*^a^* | 0.04 | 0.50*^b^* | 0.04 | 0.56*^ab^* | 0.04 | 0.090 |
| +731 | Exon 2 | 1 | 0.33^ab^ | 0.06 | 0.20^a^ | 0.06 | 0.38^b^ | 0.06 | **0.028** |
| +735 | Exon 2 | 1 | 0.42^a^ | 0.06 | 0.31^a^ | 0.06 | 0.57^b^ | 0.06 | **8.3·10^-4^** |
|  |  | 4 | 0.75^a^ | 0.03 | 0.87^b^ | 0.03 | 0.79^ab^ | 0.03 | **0.010** |
| +1032 | Intron 2 | 4 | 0.32^a,^*^a^* | 0.02 | 0.41^b,^*^ab^* | 0.02 | 0.38^ab,^*^b^* | 0.02 | **0.006** |

Shown is the influence of dietary zinc concentrations on the methylation status of CpGs in regulatory regions of the *ZIP4* gene in the epithelium of the jejunal intestine of piglets fed the different zinc diets. Abbreviations: LZn, low dietary zinc = 57 mg zinc/kg feed, n_Week1_ = 8, n_Week4_ = 9; NZn, normal dietary zinc = 164 mg zinc/kg feed, n_Week1_ = 8, n_Week4_ = 10; HZn, high dietary zinc = 2,425 mg zinc/kg feed, n_Week1_ = 8, n_Week4_ = 10. ^a,b^Labelled means with different letters in a row are significantly different (*p* < 0.05). *^a.b^*Labelled means with different letters in a row are different by trend (*p* ≤ 0.1).
