# Supplementary material for: The Zinc Concentration in the Diet and the Length of the Feeding Period Affect the Methylation Status of the ZIP4 Zinc Transporter Gene in Piglets
Source: PLoS One. 2015 Nov 23;10(11):e0143098. doi: 10.1371/journal.pone.0143098 (PMC4658085; doi:10.1371/journal.pone.0143098)
Supplement: S3 Table — Shown are the Spearman’s correlation coefficients between the methylation of CpGs of the ZIP4 gene and expression of the transcripts ZIP4-201 and ZIP4-001 over all diet groups for feeding periods of one (1) or four (4) weeks or both periods together (1+4) in the jejunal epithelium of the small intestine of piglets fed the different zinc diets. (DOCX) [file pone.0143098.s005.docx]

S3 Table. Correlation of *ZIP4* methylation with the expression of the long transcripts in the jejunal epithelium.

| CpG position | *ZIP4* gene region | Feeding period, *weeks* | | Coefficient | *p*-value | n |
| --- | --- | --- | --- | --- | --- | --- |
| -214 |  | 1 | | -0.34 | < 0.196 | 16 |
|  | 5´-Region | 4 | | -0.19 | < 0.312 | 29 |
|  |  | 1+4 | | **-0.41** | **0.005** | 45 |
| -153 |  | 1 | | -0.47 | < 0.066 | 16 |
|  | 5´-Region | 4 | | -0.01 | < 0.977 | 29 |
|  |  | 1+4 | | **-0.34** | **< 0.023** | 45 |
| -147 and -127 |  | 1 | | -0.44 | 0.092 | 16 |
|  | 5´-Region | 4 | | -0.04 | 0.829 | 29 |
|  |  | 1+4 | | **-0.37** | **< 0.013** | 45 |
| -147 |  | 1 | | -0.48 | 0.059 | 16 |
|  | 5´-Region | 4 | | -0.09 | 0.638 | 29 |
|  |  | 1+4 | | **-0.40** | **0.007** | 45 |
| -127 |  | 1 | -0.35 | | < 0.196 | 16 |
|  | 5´-Region | 4 | -0.04 | | 0.839 | 29 |
|  |  | 1+4 | **-0.32** | | **0.035** | 44 |
| -114 |  | 1 | | -0.43 | < 0.100 | 16 |
|  | 5´-Region | 4 | | 0.04 | 0.837 | 29 |
|  |  | 1+4 | | **-0.32** | **< 0.031** | 45 |
| -13 and -6 |  | 1 | | -0.40 | < 0.140 | 15 |
|  | 5´-Region | 4 | | -0.21 | < 0.278 | 28 |
|  |  | 1+4 | | **-0.46** | **< 0.002** | 43 |
| -13 |  | 1 | | -0.38 | 0.164 | 15 |
|  | 5´-Region | 4 | | -0.16 | 0.425 | 28 |
|  |  | 1+4 | | **-0.44** | **0.003** | 43 |
| -6 |  | 1 | | -0.41 | 0.126 | 15 |
|  | 5´-Region | 4 | | -0.29 | < 0.137 | 28 |
|  |  | 1+4 | | **-0.48** | **0.001** | 43 |
| +59 |  | 1 | | -0.45 | < 0.128 | 13 |
|  | Exon 1 | 4 | | -0.07 | 0.706 | 29 |
|  |  | 1+4 | | **-0.35** | **0.025** | 42 |
| +73 |  | 1 | | -0.49 | 0.066 | 15 |
|  | Exon 1 | 4 | | 0.02 | 0.923 | 29 |
|  |  | 1+4 | | **-0.32** | **0.033** | 44 |
| +405 |  | 1 | | -0.35 | 0.178 | 16 |
|  | Intron 1 | 4 | | -0.32 | < 0.089 | 29 |
|  |  | 1+4 | | **-0.46** | **< 0.002** | 45 |
| +731 to +767 |  | 1 | | **-0.50** | **< 0.049** | 16 |
|  | Exon 2 | 4 | | -0.08 | 0.669 | 29 |
|  |  | 1+4 | | **-0.41** | **< 0.005** | 45 |
| +735 |  | 1 | | **-0.65** | **< 0.007** | 16 |
|  | Exon 2 | 4 | | 0.04 | 0.843 | 29 |
|  |  | 1+4 | | **-0.39** | **0.008** | 45 |
| +767 |  | 1 | | **-0.50** | **0.049** | 16 |
|  | Exon 2 | 4 | | -0.28 | < 0.137 | 29 |
|  |  | 1+4 | | **-0.48** | **8·10^-4^** | 45 |
| +791 |  | 1 | | **-0.61** | **< 0.012** | 16 |
|  | Exon 2 | 4 | | -0.22 | < 0.248 | 29 |
|  |  | 1+4 | | **-0.46** | **0.001** | 45 |
| +795 |  | 1 | | -0.42 | < 0.105 | 16 |
|  | Exon 2 | 4 | | **-0.37** | **< 0.050** | 29 |
|  |  | 1+4 | | **-0.51** | **3·10^-4^** | 45 |
| +816 |  | 1 | | -0.22 | 0.411 | 16 |
|  | Exon 2 | 4 | | -0.24 | 0.213 | 29 |
|  |  | 1+4 | | **-0.44** | **0.002** | 45 |
| +987 |  | 1 | | -0.41 | 0.115 | 16 |
|  | Intron 2 | 4 | | -0.23 | 0.222 | 29 |
|  |  | 1+4 | | **-0.43** | **0.003** | 45 |
| +1062 |  | 1 | | -0.39 | 0.191 | 13 |
|  | Intron 2 | 4 | | -0.02 | < 0.917 | 27 |
|  |  | 1+4 | | **-0.33** | **< 0.035** | 40 |

Shown are the Spearman’s correlation coefficients between the methylation of CpGs of the *ZIP4* gene and expression of the transcripts ZIP4‑201 and ZIP4‑001 over all diet groups for feeding periods of one (1) or four (4) weeks or both periods together (1+4) in the jejunal epithelium of the small intestine of piglets fed the different zinc diets.
