# Supplementary material for: The Zinc Concentration in the Diet and the Length of the Feeding Period Affect the Methylation Status of the ZIP4 Zinc Transporter Gene in Piglets
Source: PLoS One. 2015 Nov 23;10(11):e0143098. doi: 10.1371/journal.pone.0143098 (PMC4658085; doi:10.1371/journal.pone.0143098)
Supplement: S4 Table — Shown are the Spearman’s correlation coefficients between the methylation of CpGs of the ZIP4 gene and expression of the transcript ZIP4-004 over all diet groups for feeding periods of one (1) or four (4) weeks or both periods together (1+4) in the jejunal epithelium of the small intestine of piglets fed the different zinc diets. (DOCX) [file pone.0143098.s006.docx]

S4 Table. Correlation of *ZIP4* methylation with the expression of the short transcript in the jejunal epithelium.

| CpG position | *ZIP4* gene region | Feeding period, *weeks* | Coefficient | *p*-value | n |
| --- | --- | --- | --- | --- | --- |
| +731 to +767 |  | 1 | **-0.57** | **< 0.020** | 16 |
|  | Exon 2 | 4 | -0.09 | < 0.657 | 29 |
|  |  | 1+4 | 0.04 | 0.819 | 45 |
| +743 |  | 1 | -0.48 | 0.059 | 16 |
|  | Exon 2 | 4 | **-0.50** | **0.005** | 29 |
|  |  | 1+4 | -0.11 | < 0.459 | 45 |

Shown are the Spearman’s correlation coefficients between the methylation of CpGs of the *ZIP4* gene and expression of the transcript ZIP4‑004 over all diet groups for feeding periods of one (1) or four (4) weeks or both periods together (1+4) in the jejunal epithelium of the small intestine of piglets fed the different zinc diets.
