# Supplementary material for: The Zinc Concentration in the Diet and the Length of the Feeding Period Affect the Methylation Status of the ZIP4 Zinc Transporter Gene in Piglets
Source: PLoS One. 2015 Nov 23;10(11):e0143098. doi: 10.1371/journal.pone.0143098 (PMC4658085; doi:10.1371/journal.pone.0143098)
Supplement: S5 Table — (DOCX) [file pone.0143098.s007.docx]

**S5 Table. Transcription factors which binding sites overlap with analysed CpG positions.**

| Transcription factor | Full name | CpG positions |
| --- | --- | --- |
| AML-1 | Acute Myeloid Leukemia 1 Protein | 5´‑Region: ‑264, ‑251 |
| ARNT | Aryl Hydrocarbon Receptor Nuclear | 5´‑Region: ‑2117, ‑44, ‑42 |
|  | Translocator | Exon 1: +115, +117, +122, +154 |
|  |  | Intron 1: +383 |
|  |  | Exon 2: +791, +816 |
|  |  | Intron 2: +1001 |
| Bsap | B‑Cell Lineage Specific Activator | 5´‑Region: ‑264, ‑251, ‑202, ‑189 |
|  | Protein | Intron 1: +269, +274 |
| c-REL | Proto‑Oncogene C‑Rel | Intron 1: +335 |
| CREB 1 | CAMP Responsive Element | Exon 1: +165, +171 |
|  | Binding Protein 1 | Exon 2: +816 |
| E2F | E2F Transcription Factor 1 | 5´‑Region: ‑175 |
| E47 | Transcription Factor 3 | Exon 1: +176 |
|  |  | Intron 1: +405 |
| HAND1 | Heart And Neural Crest Derivatives | Exon 1: +176 |
|  | Expressed 1 | Intron 1: +405 |
| Max | MYC Associated Factor X | 5´‑Region: ‑2117 |
|  |  | Exon 1: +115, +117, +122 |
|  |  | Intron 1: +383 |
|  |  | Intron 2: +1001 |
| n-MYC | Neuroblastoma MYC Oncogene | 5´‑Region: ‑2117, ‑44, ‑42 |
|  |  | Exon 1: +115, +117, +122, +154 |
|  |  | Intron 1: +383 |
|  |  | Exon 2: +791, +816 |
|  |  | Intron 2: +1001 |
| SAP-1 | Serum Response Factor Accessory Protein 1 | 5´‑Region: ‑2205 |
| TEF-1 | Transcriptional Enhancer Factor 1 | 5´‑Region: ‑175 |
| USF | Upstream Transcription Factor 1 | 5´‑Region: ‑2117 |
|  |  | Exon 1: +115, +117, +122, +154 |
|  |  | Intron 1: +383 |
|  |  | Exon 2: +791, +816 |
|  |  | Intron 2: +1001 |
